# The Role of (H_2_O)_1-2_ in the CH_2_O + ClO Gas-Phase Reaction

**DOI:** 10.3390/molecules23092240

**Published:** 2018-09-03

**Authors:** Junyao Li, Narcisse T. Tsona, Lin Du

**Affiliations:** Environment Research Institute, Shandong University, Binhai Road 72, Qingdao 266237, China; lijunyaoeri@mail.sdu.edu.cn (J.L.); tsonatch@sdu.edu.cn (N.T.T.)

**Keywords:** radical-molecule reaction, catalytic effect, hydrogen transfer, reaction mechanism, reaction kinetics

## Abstract

Mechanism and kinetic studies have been carried out to investigate whether one and two water molecules could play a possible catalytic role on the CH_2_O + ClO reaction. Density functional theory combined with the coupled cluster theory were employed to explore the potential energy surface and the thermodynamics of this radical-molecule reaction. The reaction proceeded through four different paths without water and eleven paths with water, producing H + HCO(O)Cl, Cl + HC(O)OH, HCOO + HCl, and HCO + HOCl. Results indicate that the formation of HCO + HOCl is predominant both in the water-free and water-involved cases. In the absence of water, all the reaction paths proceed through the formation of a transition state, while for some reactions in the presence of water, the products were directly formed via barrierless hydrogen transfer. The rate constant for the formation of HCO + HOCl without water is 2.6 × 10^−16^ cm^3^ molecule^−1^ s^−1^ at 298.15 K. This rate constant is decreased by 9−12 orders of magnitude in the presence of water. The current calculations hence demonstrate that the CH_2_O + ClO reaction is impeded by water.

## 1. Introduction

Volatile organic compounds (VOCs), originating from both anthropogenic and naturally occurring sources, are numerous and ubiquitous in the atmosphere. Formaldehyde (CH_2_O) is one of the most significant and highly reactive VOCs in the atmosphere at low levels, which can be discharged directly from vehicle exhaust and industrial processes in urban areas, and indirectly as a product of photochemical oxidation of methane and nonmethane hydrocarbons [1,2]. CH_2_O has a large accumulation in air due to its thermodynamic stability and can cause severe environmental pollution. CH_2_O is the most abundant carbonyl compound with the proportion of 70–80% in the atmosphere, and it is known to participate in many crucial chemical reactions [3,4,5].

Chlorine monoxide (ClO) radicals play a key role in the depletion of ozone by Freons in the atmosphere [6]. ClO species are mainly derived from the photolysis of CFCs in the atmosphere and sea spray [7]. Experimentally, Yuasa et al. investigated the combustion process of NH_4_ClO_4_ and reported that ClO radicals were produced near the surface of a combustor at high temperature at identical concentration as the OH radicals [8]. ClO radicals have long been known to react with various atmospheric species, including CH_3_O_2_ [9], HO_2_ [10], NH_2_ [11] producing active halogen atoms or reservoir species. The molecule-radical reaction between CH_2_O and ClO can potentially form different products as follows:

CH_2_O + ClO → HCO + HOCl (1a)

→ H + HC(O)OCl(1b)

→ Cl + HC(O)OH(1c)

→ HCl + HCOO(1d)

The CH_2_O + ClO reaction may act as a sink for CH_2_O by forming stable products such as HOCl and HCO, in which HOCl is a reservoir for ClO_x_ species [12,13]. Therefore, the studied reaction is likely to play a vital role in atmospheric chemistry, though it has been weakly explored. The kinetics of this reaction have been explored experimentally using the discharge flow electron paramagnetic resonance technique and the upper limit of the rate constant was found to be 10^−15^ cm^3^ molecule^−1^ s^−1^ at 298 K [14]. Similarly, in 1997, DeMore et al. reported the evaluated rate constant of CH_2_O + ClO reaction compiled by NASA Panel for Data evaluation to be less than 1.0 × 10^−15^ cm^3^ molecule^−1^ s^−1^ at 298 K [15]. Using ab initio molecular orbital theory at the QCISD(T)/6-311G(2d,2p)//B3LYP-D3/6-311G(d, p) level of theory, Tian et al. investigated the CH_2_O + ClO reaction and reported that the most favorable channel proceeded through the formation of HOCl + HCO via H abstraction at the investigated temperature [16]. The calculated overall rate constant was 5.8 × 10^−17^ cm^3^ molecule^−1^ s^−1^ in their study, which was consistent with the upper limit value of the results of Poulet et al. [14]. While water could potentially affect this reaction, previous studies have only focused on the water-free system.

As one of the most important atmospheric components related to earth radiation and global heat exchange, the effect of water has widely been a concern in unimolecular and bimolecular reactions [17,18]. There exists a large amount of water and water clusters in the atmosphere. Water is known to possess the ability of forming hydrogen bonds with polar molecules and reactive radicals in the atmosphere. Water could play a role as donor and acceptor for hydrogen bonds. Thus, it can easily form ring-like structures with other species, making them very stable [19]. The atmospherically relevant complexes, including water-molecule complexes [20,21,22,23,24,25,26,27] and water-radical complexes [20,21,28,29,30,31,32,33,34,35] have been paid special attention. These complexes can markedly affect the atmospheric chemical processes, such as the change of the photochemical characteristics of the atmospheric species, and the formation of aerosol particles. Hydrogen bonding controls many chemical reactions in nature. Recently, a large amount of investigations focusing on the role of water in the gas-phase reactions have been performed [28,36,37,38,39,40,41,42]. The possible effect of one water molecule on lowering the energy barrier of relevant oxidation reactions of VOC, such as formaldehyde, glyoxal, acetone and acetaldehyde, has been investigated in many experimental and theoretical studies [3,39,42,43,44,45,46,47,48,49,50]. Furthermore, some studies have shown that water dimer, with an atmospheric concentration of 9.0 × 10^14^ molecules cm^−3^ at 298 K [35,51,52,53], can also play a significant catalytic role in the H abstraction reactions. Therefore, the effect of water on the CH_2_O + ClO reaction needs to be studied further to obtain a comprehensive knowledge of this atmospheric process. In the lower troposphere, weakly bonded complexes are relatively unstable due to the high temperature and their experimental study becomes rather difficult. Under these circumstances, theoretical calculations can be the indicated tool to investigate such species. To the best of our knowledge, there are no theoretical and experimental works performed on the formaldehyde and ClO reaction to investigate the role of water.

In this paper, detailed pathways for the CH_2_O + ClO reaction in the water-free and water-involved cases have been investigated theoretically, aiming at elucidating the reaction mechanism and clarifying the effect of water on the basis of the detailed potential energy surfaces. The thermodynamics and kinetics of all investigated pathways are explored, and the atmospherically most relevant paths are highlighted. The obtained results help to gain a deeper insight into the water effect on the rate constants of representative atmospheric reactions between VOCs and reactive halogen species complexes.

## 2. Computational Methods 

The quantum chemistry calculations were carried out to investigate the CH_2_O + ClO reaction. The geometric parameters on the potential energy surface (PES) including all the stationary points were fully optimized by using the B3LYP functional with the aug-cc-pVTZ basis set [54,55,56]. The Grimme’s D3 correctional scheme was used to account for dispersion [57]. The B3LYP functional has been extensively used in theoretical studies focusing on hydrogen abstraction reactions, and exhibited results in reasonable agreement with experiments [16,58,59]. At this theory level, harmonic vibrational frequencies were also calculated to identify for local minima and transition states and to provide the zero-point vibrational energy (ZPE) correction. The connection between each transition state and the corresponding minima has been confirmed by performing intrinsic reaction coordinate (IRC) calculations [60]. To obtain more reliable energetics, single point energy calculations were performed on B3LYP-D3/aug-cc-pVTZ optimized geometries using the CCSD(T)/aug-cc-pVTZ method [61,62]. All geometry optimizations and vibrational frequency analysis were performed using the Gaussian 09 program [63].

To estimate the reactions kinetics, the rate constants of investigated reactions were calculated using the harmonic transition state theory coupled with steady-state approximation [64]. Equation (2) shows that the title reaction, regardless of the presence of water, starting from the formation of an intermediate before releasing the final products.

(2) $$CH_{2}O+ClO\overset{k_{1}}{\underset{k_{-1}}{⇄}}\mathrm{Intermediate}\overset{k_{2}}{\to }\mathrm{Products}$$

where *k*_1_ and *k*_−1_ are the collision rate constant of CH_2_O and ClO, and the dissociation rate constant of the intermediate, respectively. *k*_2_ denotes the unimolecular rate constant of the reaction from the intermediate to the products.

Assuming that for a given reaction path the intermediate is in equilibrium with the reactants, the steady-state analysis of the intermediate led to the overall rate constant of the investigated pathway given by

(3) $$k=\frac{k_{1}\;}{k_{-1}+k_{2}}k_{2}$$

If *k*_2_ << *k*_−1_, *k* can be expressed as

(4) $$k=\frac{k_{1}\;}{k_{-1}}k_{2}=K_{eq}k_{2}$$

where *K*_eq_ denotes the equilibrium constant for the formation of pre-reaction complex. *K*_eq_ is represented by

(5) $$K_{eq\;}=\frac{1}{\rho_{0}}\exp(-\frac{\Delta G}{RT})$$

*ρ*_0_ is the standard density, having a value of 2.4 × 10^19^ molecules cm^−3^ at 1 atm and 298 K. Δ*G* is the Gibbs free energy of formation of the pre-reaction complex, *R* is the molar gas constant, and *T* is the absolute temperature.

*k*_2_ is determined by the harmonic transition state theory [65]:

(6) $$k_{2}=\frac{\Pi \nu_{react\;}}{\Pi \nu_{TS}}\times \exp(-\frac{\Delta E}{RT})$$

*ν_react_* are the harmonic frequencies for the pre-reaction complex and *ν_TS_* are the harmonic frequencies for the transition state, in which only the normal vibrational frequencies are considered. Δ*E* is the energy barrier between the pre-reaction intermediate and the products. 

It is noteworthy that the paths starting from the pre-reaction complexes could be complex, with more than one transition state configuration. In these cases, *k*_2_ is calculated according to the canonical unified statistical model described by Equation (7) [66,67,68].

(7) $$\frac{1}{k_{2T\;}}=\frac{1}{k_{TS-IMx}}+\frac{1}{\Sigma k_{TSi}}$$

where *k*_2T_ is the total unimolecular rate constant, *k_TS-IMx_* denotes the rate constant of the isomerization of the initially formed complex *x*, *Σk_TS__i_* denotes the sum of rate constants of the successive transition states starting from the isomerization product.

## 3. Results and Discussion

**Figure 1:**
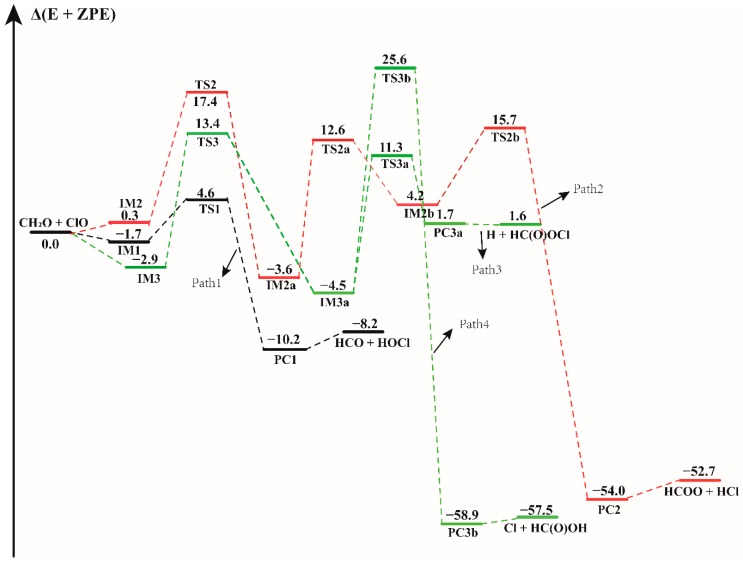
The energy profile of the CH_2_O + ClO water-free reaction. Energies (in kcal mol^−1^) are calculated at the CCSD(T)/aug-cc-pVTZ//B3LYP-D3/aug-cc-pVTZ level.

**Figure 2:**
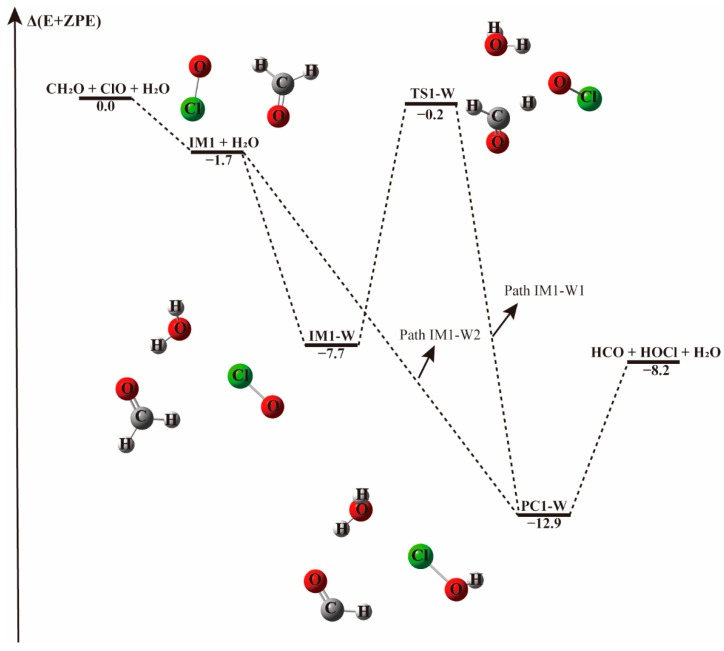
The energy profile of the CH_2_O + ClO water-free reaction occurring through the IM1 + H_2_O pathway. Energies (in kcal mol^−1^) are calculated at the CCSD(T)/aug-cc-pVTZ//B3LYP-D3/aug-cc-pVTZ level.

**Figure 3:**
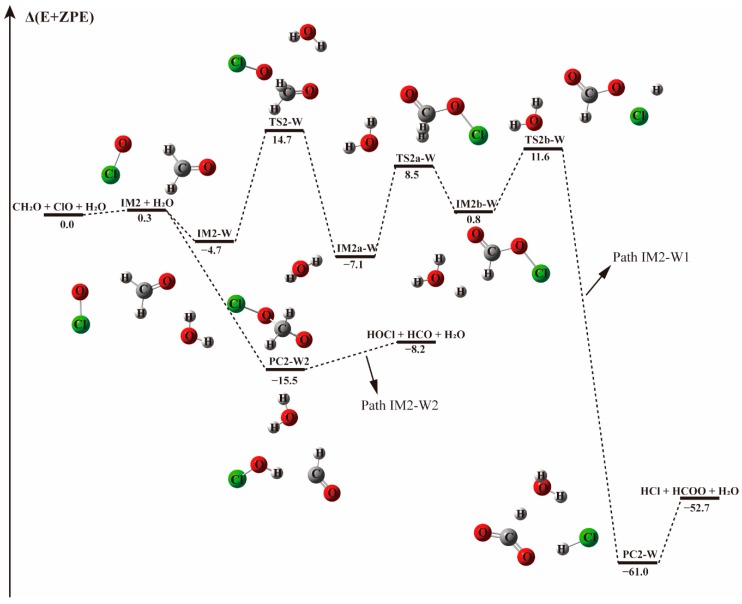
The energy profile of the CH_2_O + ClO water-fee reaction occurring through IM2 + H_2_O pathway. Energies (in kcal mol^−1^) are calculated at the CCSD(T)/aug-cc-pVTZ//B3LYP-D3/aug-cc-pVTZ level.

**Figure 4:**
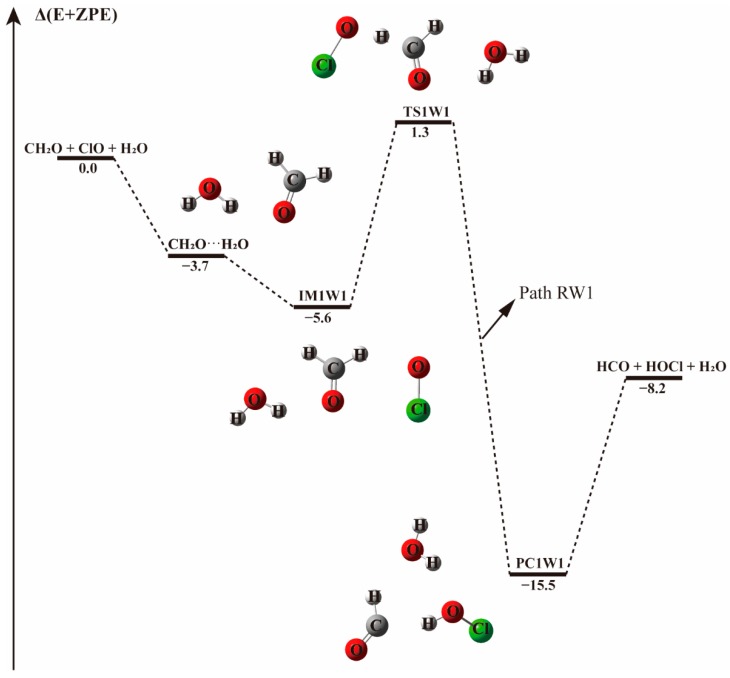
The energy profile of the CH_2_O + ClO water-involved reaction occurring through the CH_2_O···H_2_O + ClO pathway. Energies (in kcal mol^−1^) are calculated at the CCSD(T)/aug-cc-pVTZ//B3LYP-D3/aug-cc-pVTZ level.

**Figure 5:**
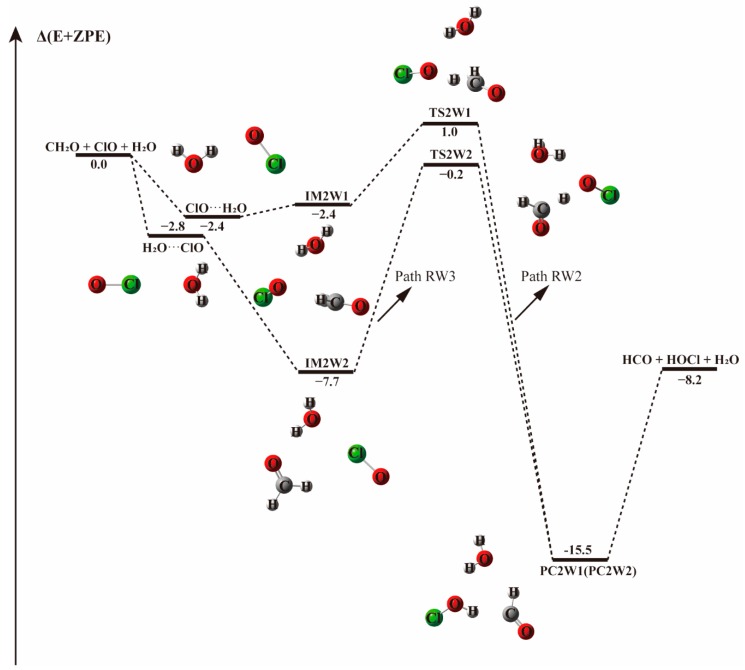
The energy profile of the CH_2_O + ClO water-assisted reaction occurring through ClO···H_2_O + CH_2_O pathway. Energies (in kcal mol^−1^) are calculated at the CCSD(T)/aug-cc-pVTZ//B3LYP-D3/aug-cc-pVTZ level.

**Figure 6:**
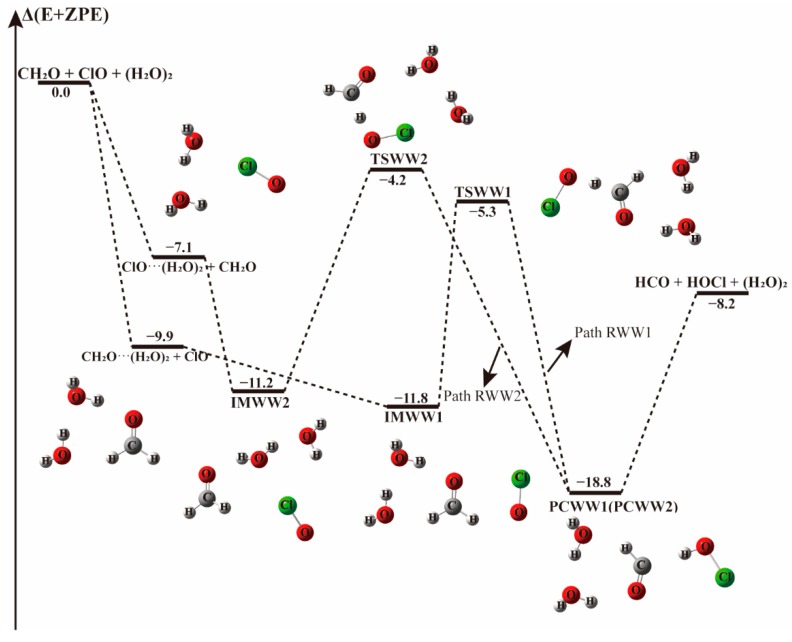
The energy profile of the CH_2_O + ClO water-involved reaction occurring through CH_2_O···(H_2_O)_2_ + ClO and ClO···(H_2_O)_2_ + CH_2_O pathways. Energies (in kcal mol^−1^) are calculated at the CCSD(T)/aug-cc-pVTZ//B3LYP-D3/aug-cc-pVTZ level.

Electronic structure calculations on the CH_2_O + ClO reactions without and with water have been performed at the CCSD(T)/aug-cc-pVTZ//B3LYP-D3/aug-cc-pVTZ level of theory. Prior to these calculations, hydrogen-bonded CH_2_O···H_2_O, ClO···H_2_O, H_2_O···ClO, CH_2_O···(H_2_O)_2_ and ClO···(H_2_O)_2_ complexes were optimized to start the reactions involving water, and their relevant conformations are shown in Figure S1. The potential energy surfaces of the CH_2_O + ClO reactions in the absence and in the presence of water are presented in Figure 1, Figure 2, Figure 3, Figure 4, Figure 5 and Figure 6. Finally, the rate constant for each reaction path has been computed within the temperature range 216.69–298.15 K (corresponding to the temperatures in the 0–12 km altitude). All transition states involved in these reactions are labeled by prefix TS, the pre-reaction and post-reaction complexes are denoted as IM and PC, respectively. The inclusion of “W” and “WW” in these notations denote the presence of water monomer and water dimer, respectively.

### 3.1. Mechanism of the CH_2_O + ClO Reaction in the Absence of Water Vapor

In the CH_2_O + ClO water-free reaction, four reaction pathways have been found to proceed from three binary complexes (IM1, IM2 and IM3) in the entrance channel, forming four different products as depicted in Figure 1. The configurations of all the pre-reaction complexes, transition states, and post-reaction complexes are displayed in Figure S2. All these pathways proceed through the formation of at least one transition state configuration towards the formation of the final products. The formation energies with ZPE correction (Δ(E + ZPE)), enthalpies (ΔH) and Gibbs free energies (ΔG) of all the species involved in these reactions, relative to the initial reactants are listed in Table 1. Similar to the previous computational results, the first step of the formation of HOCl + HCO involves the formation of a pre-reaction molecular complex IM1 located at 1.7 kcal mol^−1^ electronic energy below the separate reactants. The distance between the H atom of CH_2_O and O atom of ClO in this complex is 2.184 Å, which is in moderate agreement with the intermoiety distance of 2.022 Å in the research of Tian et al. [16]. For the formation of HOCl molecule and HCO fragment, the calculated results show that this is a H abstraction reaction. With ClO approaching the H atom of CH_2_O, the product complex PC1 is formed via a H-abstraction by ClO in the transition state TS1. As displayed in Figure 1 and Table 1, TS1 lies 4.6 kcal mol^−1^ above the reactants, which is in reasonable agreement with the value of 5.0 kcal mol^−1^ reported by Tian et al. and with the experimental result, which indicated that the energy barrier should be higher than 4.2 kcal mol^−1^ [14,16]. The difference in these energy values results from the difference in the techniques used by these studies. The energy of TS1 relative to the IM1 complex is 6.2 kcal mol^−1^. The overall Gibbs free energy (ΔG) change of this reaction has been calculated at 1 atm and 298.15 K to be −11.31 kcal mol^−1^, demonstrating that this reaction path is thermodynamically feasible. The formation of PC1 is exothermic by 10.2 kcal mol^−1^, in agreement with the experimental value of 11.2 kcal mol^−1^ and the previous theoretical result of 9.7 kcal mol^−1^ [14,16].

Beginning with the IM2 complex, the reaction firstly proceeds through the TS2 isomerization transition state with an electronic energy of 17.4 kcal mol^−1^ relative to the reactants. The binary IM2a complex forms via the O atom of the ClO radical attacking the C atom of the CH_2_O. In IM2a, the distance between the C atom and the O atom of ClO is 1.443 Å, which is consistent with the bond length of 1.441 Å in the previous result [16]. Starting from IM2a, the following step proceeds through a transition state TS2a with the breaking of the C-H bond, resulting in the formation of another intermediate IM2b (H + HC(O)OCl) and then transforming to the more stable HCOO + HCl products via the transition state TS2b. The relative energy of the transition states TS2a and TS2b are 16.2 and 11.6 kcal mol^−1^, respectively. Path 2 is exothermic by 52.7 kcal mol^−1^.

There are two other isomers (IM3a and IM4a) of IM2a, which are formed via rotation around the OC-ClO bond, having the same energy of −4.5 kcal mol^−1^ and similar electronic structures. As shown in Figure S2, they are enantiomers about the O-C-O plane. We found that they possess nearly identical behavior in the subsequent reaction pathway. Therefore, only the formation and reaction of complex IM3a are discussed in the following section. IM3a is the isomerization product of IM3, and is formed by overcoming the barrier height of 16.3 kcal mol^−1^. Beginning with IM3a, the reaction proceeds through formation of two different transition states, TS3a and TS3b, and decompose into different final products. One is the formation of H + HC(O)OCl via the TS3a transition state with an energy barrier height of 15.7 kcal mol^−1^. Through formation of the TS3b transition state, IM3a forms Cl + HC(O)OH by overcoming a much higher barrier height, 30.1 kcal mol^−1^. In this process, the breaking Cl-O bond is elongated by 0.746 Å along with the H atom approaching the O atom and forming the O-H bond. Compared to the formation of H + HC(O)OCl, the Cl + HC(O)OH products formed from the IM3a complex could be neglected due to the high energy barrier. However, the reaction path with lower energy barrier (Path 3) is still less favorable than the H-abstraction path (Path 1). The calculated results shown in Figure 1 indicate that the formation of HCO + HOCl is the most favorable path in the CH_2_O + ClO reaction without water. Obviously, our theoretical results are in accordance with previous experimental [14] and theoretical conclusions [16].

### 3.2. Mechanism of the CH_2_O + ClO Reaction in the Presence of Water Vapor

In order to evaluate the role of water in the CH_2_O + ClO atmospheric reaction, we explored the mechanisms and energetics of the systems with one and two water molecules. In the presence of one water molecule, considering the low probability of a termolecular collision, all possible binary complexes colliding with the third species are considered in the entrance channel. Six binary complexes were found in the initial steps of the CH_2_O + ClO + H_2_O reaction. On the one hand, either CH_2_O or ClO interacts with a water molecule via hydrogen bond or halogen bond to form the CH_2_O···H_2_O, ClO···H_2_O, H_2_O···ClO binary complexes with relative formation electronic energies of −3.7, −2.4 and −2.8 kcal mol^−1^, respectively, that could further form ternary complexes with the third species. For the interaction between CH_2_O and H_2_O, only one configuration was identified, where the CH_2_O···H_2_O complex is stabilized by one hydrogen bond formed between the O atom of CH_2_O and one H atom of water. However, for the interaction between ClO and H_2_O, one hydrogen bonded complex ClO···H_2_O in which water acts as hydrogen bond donor, and one halogen bonded complex H_2_O···ClO was obtained as depicted in Figure S1. On the other hand, interactions between the IM1, IM2 and IM3 complexes discussed above and water were considered. The three-body intermediates formed between binary complexes and corresponding third species further overcome transition states energy barriers to form post-reactive complexes and then release the products. We found that the products remain unchanged when a water molecule is present. Nine different reaction pathways were investigated.

Two water molecules could interact with each other via a hydrogen bond forming the water dimer. Some studies have shown that water dimer can have significant catalytic effects in the hydrogen abstraction reactions [35,51,52,59,69,70,71,72,73,74]. Due to its high concentration of 3.3 × 10^14^ molecules cm^−3^ at 298 K [35], water dimer can effectively interact with a third species, CH_2_O or ClO, before proceeding further. This highlights that the complexes with water dimer must be taken into account to get a broader knowledge of the impact of ubiquitous water.

It is worthy to note that in the water monomer and water dimer cases, water could act as a “bridge” in the H atom abstraction from CH_2_O by ClO. These paths involve a double hydrogen transfer mechanism, which needs to surmount a much higher energy barrier, as mentioned in the previous study of Zhang et al. [75]. Taking one path starting from ClO···H_2_O + CH_2_O as an example, the relevant geometrical configurations and potential energy surface are displayed in Figures S4 and S5, respectively. The corresponding energetics are listed in Table 2. The higher barrier of the “water bridge” mechanism is in agreement with the results obtained for the HO + HOCl [68] and H_2_S + HO reactions [35]. Thus, we focused our attention only on the direct H-abstraction paths in the remainder of the discussions.

#### 3.2.1. Reaction Between (H_2_O)_1–2_ and IM1/IM2/IM3 Complexes−

The effect of water monomer and water dimer was investigated by first considering the interaction between the IM1/IM2/IM3 complexes and water/water dimer. The optimized geometries of IM1, IM2, and IM3 complexes are exhibited in Figure S2. The schematic energy diagrams of the channels with one and two water molecules are illustrated in Figure 2, Figure 3, Figures S3 and S6. Two reaction mechanisms, the direct and indirect H abstraction, have been found depending on the collisional directions. Some orientations of water addition resulted in the barrierless formation of HOCl + HCO.

When a single water molecule was added into the IM1 system, two kinds of hydrogen bonds were formed. Water could form hydrogen bonds with the O atom of both ClO and CH_2_O. Water addition to the IM1 system forming hydrogen bond with the O atom of CH_2_O leads to the formation of a ternary complex denoted IM1-W, shown in Figure 2. This complex is stable due to the formation of hydrogen bond between the H atom of water and O atom of CH_2_O, and a halogen bond between the O atom of water and Cl atom of ClO, with a binding electronic energy of −7.7 kcal mol^−1^. In the formation of this complex, water monomer serving as a third body on the potential energy surface of the naked reaction, stabilizes the pre-reaction complex. Similar to the H-abstraction reaction without water, beginning with IM1-W, the O atom of ClO abstracts the H atom of CH_2_O followed by the elongation of the C-H bond from 1.102 Å in IM1-W to 1.238 Å in TS1-W. Moreover, the pre-reaction complex IM1-W and the transition state TS1-W are more stabilized than the corresponding water-free complex IM1 and TS1 as can be seen from their respective Gibbs free energy changes. The barrier height for this process is 7.5 kcal mol^−1^ in terms of electronic energy, 1.3 kcal mol^−1^ higher than that found in the water-free path. It seems that water increase the activated barrier of H-abstraction transition state by stabilizing the pre-reaction complex.

However, when a water molecule happens to attacks IM1 from an interaction distance of 2.91 Å, the H atom of CH_2_O could be directly abstracted by ClO and result in the barrierless formation of the product complex, as indicated in Figure 2, Path IM1-W2. This means that, depending on the incoming direction of water, the H-abstraction in the CH_2_O + ClO reaction forming HOCl^+^ HCO can be highly facilitated. In terms of the Gibbs free energy change, as listed in Table 1 and Table 2, ΔG at 298 K for the barrierless formation of HCO + HOCl via Path IM1-W2 is −3.8 kcal mol^−1^, indicating that the barrierless formation of HCO + HOCl from the CH_2_O + ClO water-assisted reaction is thermodynamically favorable at ambient temperature. Hence, the formation of HCO + HOCl is facilitated by the presence of one water molecule at these conditions.

The pre-reaction complex IM2-W is observed to be 3.0 kcal mol^−1^ less stable than IM1-W, lying 4.7 kcal mol^−1^ below the energy of reactants, as shown in Table 3. The addition of water to form IM2-W does not change the initial relative positions of ClO and CH_2_O in IM2. Their formation Gibbs free energies are 9.82 and 6.9 kcal mol^−1^, respectively, indicating that the presence of water destabilizes the IM2 complex. Starting from IM2-W, the Path IM2-W1 is similar to Path 2 of the water-free reaction channel. This channel proceeds via a stepwise mechanism, in which the O atom of ClO collides with the C atom of CH_2_O in the favorable direction to form IM2a-W via the TS2a transition state, though their energies differ slightly. IM2a-W proceeds through a unimolecular decomposition pathway firstly forming H + HC(O)OCl + H_2_O (IM2b-W), then the H atom interacts with the Cl atom of the HC(O)OCl fragment, followed by the breaking of the bond between Cl and O and finally resulting in the formation of HCOO, HCl and water. From an energetic point of view, the transition state (TS2a–W) energy for the first decomposition is 15.5 kcal mol^−1^, 0.7 kcal mol^−1^ lower that that without water. Similarly, the second transition state, TS2b-W, with the relative energy of 11.55 kcal mol^−1^, is 0.8 kcal mol^−1^ lower than in that of the corresponding water-free reaction path. Generally, the presence of water weakly affects the transition state energy. As evidenced in Figure 1 and Figure 3, the reaction paths beginning with the formation of the IM2 complex and producing HCl and HCOO (Paths 2 and IM2-W1) are likely irrelevant for the reaction between CH_2_O and ClO regarding the high energy barrier and the complexity of the reaction process.

However, upon addition of a water molecule to IM2 with a hydrogen bond formed between the Cl atom of ClO and H atom of H_2_O, instead of forming the IM2-W three-body complex, the O atom of ClO could abstract the H atom of CH_2_O directly and produce HOCl + HCO without a transition state, as depicted in Path IM2-W2. This barrierless H atom abstraction could not be observed in the reaction without water no matter how we adjusted the relative positions of the two reactants. Obviously, the presence of a single water molecule exhibits a significant catalytic effect on the HOCl + HCO formation via CH_2_O + ClO reaction.

The energy profile of the CH_2_O + ClO reaction occurring through IM3-W and producing H + HCOOCl + H_2_O and Cl + HCOOH + H_2_O is displayed in Figure S3. The energies for TS3a-W and TS3b-W are 15.9 and 29.4 kcal mol^−1^, respectively, remaining nearly unchanged compared to the corresponding paths without water (15.7 and 30.1 kcal mol^−1^, respectively). These two paths could be negligible due to their much higher energy barriers compared to the H-abstraction path. When water dimer is added to the CH_2_O···ClO complex, there exits H-abstraction channels with an energy barrier similar to the one water molecule-assisted paths (see Figure 2 and Figure 3).

The potential energy surfaces of the CH_2_O + ClO reaction occurring through IM1 + (H_2_O)_2_ and IM2 + (H_2_O)_2_ are displayed in Figure S6. First, the CH_2_O···ClO complex formed a cage-like hydrogen bonding network, IM1-2W, with water dimer, with −11.1 kcal mol^−1^ binding energy. Then the reaction progressed through TS1-2W with the H atom of CH_2_O moving to the ClO group, with an energy barrier of 7.1 kcal mol^−1^, which is lower by 0.4 kcal mol^−1^ than that of the transition state TS1-W with one water molecule. This indicates that the inclusion of a second water molecule is not very crucial in this path. The post-reactive complex PC1-2W is also a cage-like hydrogen bonding network that forms with −20.2 kcal mol^−1^ binding energy, and then decomposes to the final products HOCl and HCO. As can be observed from Figure S6, the H abstraction from CH_2_O by ClO can proceed in a barrierless manner in both the IM1 and IM2 reactions when water dimer is present, similar to the one water-catalyzed case. In the presence of water dimer, the HOCl and HCO products are formed directly without energy barrier, indicating that water dimer also plays a catalytic role on the CH_2_O + ClO reaction.

#### 3.2.2. Mechanism of the CH_2_O···(H_2_O)_1-2_ + ClO and ClO···(H_2_O)_1–2_ + CH_2_O Reactions

Starting from CH_2_O···H_2_O and ClO···H_2_O complexes, the addition of the third species leads to three possible bimolecular reactions, which are labeled as Paths RW1, RW2, and RW3 in Figure 4 and Figure 5.

In Path RW1 (see Figure 4), the bimolecular CH_2_O···H_2_O complex is formed in the first step. Water can attach to formaldehyde in one position, forming cyclic radical-molecule complex that is held by a hydrogen bond of 2.003 Å. This complex adopts a five-member-ring like configuration where the water moiety serves as hydrogen bond donor to the O on the carbonyl group of formaldehyde. When the ClO radical is added to the system, the initial CH_2_O···H_2_O configuration is hardly destroyed, the hydrogen bond formed between the hydrogen atom of H_2_O and the oxygen atom of CH_2_O remains nearly unchanged with a bond length of 2.037 Å. The ClO radical approaches the H atom of CH_2_O as in the water-free case, forming the IM1W1 molecular complex with an electronic energy change of −5.6 kcal mol^−1^. The intermoiety distance between CH_2_O···H_2_O and ClO is 2.233 Å, slightly higher than the distance between H and O atoms of 2.196 Å in the absence of water. In this path, water vapor acts as a spectator. The H-abstraction by ClO proceeds through the TS1W1 transition state with an energy barrier of 6.9 kcal mol^−1^, 0.7 kcal mol^−1^ higher than in the water-free reaction, and results in the same products. The slightly higher energy barrier in this path indicates that water may have a negligible effect on the CH_2_O···H_2_O + ClO reaction.

In the reaction paths starting from ClO and H_2_O, two different transition states are found, based on the type of bonds formed between ClO and H_2_O (see Figure 5). In path RW2, one H atom of H_2_O approaches the O atom of ClO forming a hydrogen bond in which water acts as hydrogen donor and ClO as hydrogen bond acceptor. While in Path RW3, the chlorine atom of ClO approaches the oxygen atom of water forming a halogen bond. The calculated optimized geometries of the ClO···H_2_O and H_2_O···ClO complexes are in accordance with the results of previous calculations [76,77,78,79]. Although significantly different in geometries, both configurations show similar binding energies (−2.4 and −2.8 kcal mol^−1^).

Geometries and relative energies are distinctly different for paths RW2 and RW3, but they both lead to the same final products, HCO, HOCl, and H_2_O, formed with −8.2 kcal mol^−1^ release in energy. The three-body complex IM2W2 is 5.3 kcal mol^−1^ more stable than IM2W1 due to the presence of both hydrogen bond and halogen bond in the former. The path initiated by the ClO···H_2_O binary complex is energetically favored as evidenced in Figure 5, with an energy barrier of 3.3 kcal mol^−1^, which is 2.9 kcal mol^−1^ lower than the water-free path. However, the other reaction path starting from H_2_O···ClO possesses a much higher energy barrier of 7.5 kcal mol^−1^, 4.2 kcal mol^−1^ higher than in Path RW3 and 1.3 kcal mol^−1^ higher than in the reaction without water, suggesting that the addition of a single water molecule in the ClO···H_2_O + CH_2_O reaction paths does not play crucial role in the formation of HCO + HOCl.

When there is a water dimer, as shown in Figure 6 and Table 4, in the entrance channel, two cyclic three-body complexes CH_2_O···(H_2_O)_2_ and ClO···(H_2_O)_2_ are formed with −9.9 and −7.1 kcal mol^−1^ binding energies, respectively. Subsequently, by interaction of CH_2_O···(H_2_O)_2_ with ClO or ClO···(H_2_O)_2_ with CH_2_O, two pre-reaction complexes IMWW1 and IMWW2 form with binding energies of −11.8 and −11.2 kcal mol^−1^, respectively. It is evident in Figure 6 that the initial complexes CH_2_O···(H_2_O)_2_ and ClO···(H_2_O)_2_ are stable enough that the configurations are hardly affected by the introduction of the third species. Similar to the reaction with a water molecule, starting from IMWW1 and IMWW2, with one H atom of CH_2_O moving to the adjacent ClO group, the reactions can go through transition states TSWW1 and TSWW2 to form corresponding product complexes PCWW1 and PCWW2 before releasing to the products HOCl and HCO. The configurations of the core complexes in IMWW1 and TSWW1 are similar to those in IM1W1 and TS1W1. The TSWW1 energy is 6.6 kcal mol^−1^ relative to the CH_2_O···(H_2_O)_2_ + ClO reactants for Path RWW1, which is nearly similar to the energy barrier in Path RW1(6.9 kcal mol^−1^). Exploring the Gibbs free energy changes in Table 3 and Table 4, we found that the reaction path beginning with CH_2_O···H_2_O (Path RW1) is thermodynamically more favorable than that beginning with the CH_2_O-water dimer complex (Path RWW1). In addition, the structure of IMWW2 is similar to that of IM2W2, and the energy of TSWW2 is 7.0 kcal mol^−1^ relative to IMWW2, roughly similar to the one water case, which has a barrier height of 7.5 kcal mol^−1^. It follows that similar to the one water case, the effect of water dimer on the energy barrier is not important in these paths.

### 3.3. Kinetics

The rate constant of each elementary process was determined using the harmonic transition state theory according to Equation (10). As discussed above, the formation of HCO + HOCl from CH_2_O + ClO is the major path both without and with water vapor and the rate constants for these reactions over the temperature range investigated are shown in Table 5. As evidenced from Table 5, for the reaction without water, the calculated rate constants (*k*_1_) range between 5.86 × 10^−18^ and 2.6 × 10^−16^ cm^3^ molecule^−1^ s^−1^ in the temperature range considered, in agreement with the experimental result of Poulet et al. reporting that the upper limit of the rate constant is 1.0 × 10^−15^ cm^3^ molecule s^−1^ at 298 K for the formation of HOCl + HCO [14]. According to the results, the rate constants exhibit a positive temperature dependence whether there is water or not.

For the channels with one water molecule to form HOCl + HCO, the rate constants for CH_2_O···ClO + H_2_O (*k*_IM1-W1_, Path IM1-W1), CH_2_O···H_2_O + ClO (*k*_RW1_, Path RW1), H_2_O···ClO + CH_2_O (*k*_RW3_, Path RW3) are 3–4 orders of magnitude lower than that without water, given the *k*_IM1-W1_/*k*_1_, *k*_RW1_/*k*_1_, *k*_RW3_/*k*_1_ ratios of 1.59 × 10^−4^–1.06 × 10^−3^, 2.16 × 10^−4^−5.94 × 10^−4^, 1.59 × 10^−4^−1.06 × 10^−3^, respectively, whereas the rate constant for ClO···H_2_O + CH_2_O (*k*_RW2_, Path RW2), is ~1 order of magnitude lower than those in the above paths with water within the temperature range investigated as evidenced in Table 5. We notice that the reaction rate constants are nearly the same for Paths IM1-W1 and RW3 in the whole temperature range. As shown in Figure 2 and Figure 5, the introduction of water monomer to the CH_2_O···ClO binary complex and a CH_2_O molecule to the H_2_O···ClO system results in the same three-body complex. However, with the exception of the barrierless path, the reaction rate constants for all the paths with the inclusion of water are lower than those of the water-free reaction, indicating that one water molecule would also exhibit a negative effect on the CH_2_O + ClO → HOCl + HCO reaction under tropospheric temperatures.

With the addition of water dimer, as shown in Table S6, the rate constants of Paths RWW1 and RWW2 are 6–7 orders of magnitude lower than the reaction path without water. The rate constants in both paths vary only slightly with temperature in the temperature range investigated. However, these rate constants are 3–5 orders of magnitude smaller than those of reactions in the presence of one water molecule. Therefore, the reaction channels beginning with CH_2_O···(H_2_O)_2_ and ClO···(H_2_O)_2_ could be neglected in the atmosphere.

The kinetics of a reaction channel cannot be obtained merely through the research of stationary points on the potential energy surface. Comparison of the rate constants of single reactions is not comprehensive for further insight into the effect of water on the CH_2_O + ClO reaction. It is essential to compare the effective rate constants of reactions in the presence and absence of (H_2_O)_1-2_. The rate for the water-free reaction is described by

v_1_ = *k*_1_[CH_2_O][ClO] (8)

while the rates for the HCO + HOCl formation via channels RW1, RW3, IMWW1 and IMWW2 can be written as

v_RW1_ = *k*_RW1_[CH_2_O···H_2_O][ClO] = *k*’_RW1_[CH_2_O][ClO] (9)

v_RW3_ = *k*_RW3_[H_2_O···ClO][CH_2_O] = *k*’_RW3_[CH_2_O][ClO] (10)

v_IMWW1_ = *k*_IMWW1_[CH_2_O···(H_2_O)_2_][ClO] = *k*’_IMWW1_ [CH_2_O][ClO] (11)

v_IMWW2_ = *k*_IMWW2_ [ClO···(H_2_O)_2_][CH_2_O] = *k*’_IMWW2_ [CH_2_O][ClO] (12)

The effective rate constants *k’_RW_*_1_, *k*’_RW3_, *k*’_IMWW1_ and *k*’_IMWW2_ are calculated utilizing the water concentration at given temperatures. The amount of water vapor is dependent on the temperature and reduces with altitude in general. At 100% relative humidity, 298.15 K, the calculated water vapor concentration is 7.2 × 10^17^ molecules cm^−3^ [80]. In the above equations, *k*’_RW1_ = *k*_RW1_*k*_eq1_[H_2_O], *k*’_RW3_ = *k*_RW3_*k*_eq3_[H_2_O], *k*’_IMWW1_ = *k*_IMWW1_ *k*_eq_(CH_2_O···(H_2_O)_2_)[(H_2_O)_2_] and *k*’_IMWW2_ = *k*_IMWW2_ *k*_eq_(ClO···(H_2_O)_2_)[(H_2_O)_2_] in which *k*_eq1_, *k*_eq3_, *k*_eq_(CH_2_O···(H_2_O)_2_) and *k*_eq_(ClO···**(**H_2_O)_2_) are the equilibrium constants for the formation of CH_2_O···H_2_O, H_2_O···ClO, CH_2_O···(H_2_O)_2_ and ClO···(H_2_O)_2_ complexes, respectively. The values for *k*_eq1_ and *k*_eq3_ are listed in Table S2 of the supplementary materials. Table S4 shows the effective rate constants of the reaction in the presence and absence of water under different altitudes in the earth’s atmosphere, while the ratios for the comparison of effective rate constants to the water-free rate constant *k*_1_ are given in Table S5. Table S6 displays the rate constants and corresponding effective rate constants for the reactions with a water dimer. With the increase in altitude, there is a decrease in the rate constants when the concentrations of water and water dimer are decreasing. The concentration of water dimer is more sensitive to the temperature, decreasing by five orders of magnitude as the temperature drops from 298.15 K to 216.69 K. Thus, the amounts of complexes formed between the individual reactants and water decrease with the altitude. As evidenced in Table S5, the effective rate constants for HOCl + HCO formation in the presence of water monomer are 9–12 orders of magnitude lower than that of water free case within the temperature range of 216.69–298.15 K, and the ratios show a positive temperature dependence. These results indicate that water cannot accelerate the CH_2_O + ClO gas-phase reaction to form HOCl + HCO under atmospheric conditions.

While the effective rate constants with a single water molecule are higher by 10–12 orders of magnitude than those of reactions with water dimer, both of them are much lower than the rate constant for the reaction in the absence of water, indicating that both effects of one water molecule and water dimer via CH_2_O···H_2_O + ClO, H_2_O···ClO + CH_2_O, CH_2_O···(H_2_O)_2_ + ClO and ClO···(H_2_O)_2_ + CH_2_O are negative under investigated temperatures due to the decreased reaction rate constants.

## 4. Conclusions

Both ClO and CH_2_O are important species in the polluted atmosphere. Hypochlorous acid (HOCl) produced from the CH_2_O + ClO reaction has been well known as the reservoir for ClOx in the atmosphere. Exploring the effect of water and water clusters on the energy profile and kinetics of the CH_2_O + ClO reaction is of great interest and has reached some beneficial conclusions. For the reaction without water, four reaction pathways have been found on the potential energy surface. In consistent with the results of Tian et al. [16], the formation of HOCl and HCO is the most favorable reaction path in the absence of water vapor. With the inclusion of water, four different interactions are considered at the entrance channels: CH_2_O···H_2_O + ClO, ClO···H_2_O + CH_2_O, H_2_O···ClO + CH_2_O, and CH_2_O···ClO + H_2_O. The variation of the energy barriers with the addition of water is negligible compared to the water-free path. However, we find that when one water molecule and water dimer are involved into the initially formed CH_2_O···ClO binary complex, HOCl and HCO could form without an energy barrier, indicating that water has a catalytic effect on the formation of HOCl + HCO via CH_2_O + ClO reaction. In terms of the reaction kinetics, the rate coefficients of the channels both with and without inclusion of water exhibit a positive temperature dependence, being highest at room temperature. For reactions involving transition state configurations, the effective rate constants for HCO + HOCl formation reactions in the presence of water are 9–12 orders of magnitude lower than those of reactions without water. The study of the effect of water and water dimer in this work brings further molecular insights on how water participates in gas phase reactions under atmospheric conditions. 

## Figures and Tables

**Table 1:** Electronic energies (ΔE and Δ(E + ZPE)), enthalpies [ΔH(298 K)], and Gibbs free energies [ΔG(298 K)] for the CH_2_O + ClO water-free reaction. The units of the energies are kcal mol^−1^.

System	ΔE	Δ(E + ZPE)	ΔH(298 K)	ΔG(298 K)
CH_2_O + ClO	0	0	0	0
IM1	−2.5	−1.7	−0.7	6.0
TS1	6.9	4.6	1.7	9.8
PC1	−10.0	−10.2	−10.0	−5.3
HCO + HOCl	−6.7	−8.2	−9.4	−10.9
IM2	−0.2	0.3	1.1	6.9
TS2	16.8	17.4	17.1	26.7
IM2a	−5.7	−3.6	−2.6	7.5
TS2a	14.9	12.6	9.4	19.4
IM2b	7.0	4.2	1.3	8.8
TS2b	18.3	15.7	12.5	21.4
PC2	−52.6	−54.0	−55.6	−48.2
HCOO + HCl	−49.0	−52.7	−56.0	−56.4
IM3	−3.7	−2.9	−1.9	4.6
TS3	12.8	13.4	13.0	22.6
IM3a	−6.8	−4.5	−3.2	6.9
TS3a	13.4	11.3	8.1	18.2
PC3a	4.4	1.7	−1.0	−1.0
H + HC(O)OCl	4.7	1.6	−1.6	1.5
TS3b	27.1	25.6	23.2	32.9
PC3b	−62.2	−58.9	−56.0	−48.3
Cl + HC(O)OH	−60.5	−57.5	−54.9	−52.6

**Table 2:** Electronic energies (ΔE and Δ(E + ZPE)), enthalpies [ΔH(298 K)], and Gibbs free energies [ΔG(298 K)] for the reaction beginning with IM1 + H_2_O and IM2 + H_2_O. The units of the energies are kcal mol^−1^.

System	ΔE	Δ(E + ZPE)	ΔH(298 K)	ΔG(298 K)
CH_2_O + ClO + H_2_O	0	0	0	0
IM1-W	−10.5	−7.7	**−**5.0	9.3
TS1-W	0	−0.2	−1.2	15.2
PC1-W	−14.3	−12.9	−11.2	2.2
IM2-W	−7.0	−4.7	−2.2	9.8
TS2-W	12.8	14.7	15.8	32.4
IM2a-W	−10.5	−7.1	−4.6	12.4
TS2a-W	9.2	8.5	6.8	23.8
IM2b-W	2.0	0.8	−0.5	14.4
TS2b-W	12.6	11.6	9.9	24.8
PC2-W PC2-W2	−62.3 −17.4	−61.0 −15.5	−61.1 −13.7	**−**43.2 0.4
HCl + HCOO + H_2_O	−49.0	−52.7	−56.0	−56.4

**Table 3:** Electronic energies (ΔE and Δ(E + ZPE)), enthalpies [ΔH(298 K)], and Gibbs free energies [ΔG(298 K)] for the reaction with a single water molecule occurring through CH_2_O···H_2_O + ClO and ClO···H_2_O + CH_2_O. The units of the energies are kcal mol^−1^.

System	ΔE	Δ(E + ZPE)	ΔH(298 K)	ΔG(298 K)
CH_2_O + ClO + H_2_O	0	0	0	0
CH_2_O···H_2_O + ClO	−5.6	−3.7	−2.0	4.5
ClO···H_2_O + CH_2_O	−3.5	−2.4	−1.4	4.0
H_2_O···ClO + CH_2_O	−3.6	−2.8	−1.8	3.8
IM1W1	−8.1	−5.6	−3.0	10.2
TS1W1	2.3	1.3	−0.1	14.8
PC1W1	−17.4	−15.5	−13.7	0.4
IM2W1	−4.8	−2.4	−0.1	14.9
TS2W1	1.1	1.0	−0.1	16.5
IM2W2	−10.5	−7.7	−5.0	9.3
TS2W2	0	−0.2	−1.2	15.2
IM2W3	−9.2	−6.8	−4.3	9.8
TS2W3	41.8	39.4	35.7	53.7
PC2W1(PC2W2)	−17.4	−15.5	−13.7	0.4
PC2W3	−19.7	−17.3	−15.3	−0.1
HCO + HOCl + H_2_O	−6.7	−8.2	−9.4	−10.9

**Table 4:** Electronic energies (ΔE and Δ(E + ZPE)), enthalpies [ΔH(298 K)], and Gibbs free energies [ΔG(298 K)] for the reaction occurring through CH_2_O···(H_2_O)_2_ + ClO and ClO···(H_2_O)_2_ + CH_2_O. The units of the energies are kcal mol^−1^.

System	ΔE	Δ(E + ZPE)	ΔH(298 K)	ΔG(298 K)
CH_2_O + ClO + (H_2_O)_2_	0	0	0	0
CH_2_O···(H_2_O)_2_ + ClO	−14.5	−9.9	−6.4	9.5
ClO···(H_2_O)_2_ + CH_2_O	−10.3	−7.1	−4.4	10.1
IMWW1	−16.9	−11.8	−7.5	15.4
TSWW1	−6.8	−5.3	−4.9	19.4
PCWW1	−22.6	−18.8	−15.3	6.1
IMWW2	−15.9	−11.2	−6.9	10.1
TSWW2 PCWW2	−5.4 −22.6	−4.2 −18.8	−4.0 −15.3	15.2 21.2
HCO + HOCl + 2H_2_O	−6.7	−8.2	−9.4	−10.9

**Table 5:** Rate constants (in cm^3^ molecule^−1^ s^−1^) for the CH_2_O + ClO reaction with one water molecule at different heights (h).

h (km)	T (K)		k_1_	k_IM1-W1_	k_RW1_	k_RW2_	k_RW3_
0	298.15	2.6 × 10^−16^		4.2 × 10^−20^	5.7 × 10^−20^	6.7 × 10^−21^	4.2 × 10^−20^
0	288.19	1.8 × 10^−16^		3.5 × 10^−20^	4.4 × 10^−20^	5.4 × 10^−21^	3.5 × 10^−20^
2	275.21	1.1 × 10^−16^		2.7 × 10^−20^	3.0 × 10^−20^	3.9 × 10^−21^	2.7 × 10^−20^
4	262.23	6.5 × 10^−17^		2.1 × 10^−20^	2.0 × 10^−20^	2.8 × 10^−21^	2.1 × 10^−20^
6	249.25	3. 6 × 10^−17^		1.5 × 10^−20^	1.3 × 10^−20^	2.0 × 10^−21^	1.5 × 10^−20^
8	236.27	1.8 × 10^−17^		1.1 × 10^−20^	8.0 × 10^−21^	1.3 × 10^−21^	1.1 × 10^−20^
10	223.29	8.8 × 10^−18^		7.6 × 10^−21^	4.7 × 10^−21^	8.3 × 10^−22^	7.6 × 10^−21^
12	216.69	5.9 × 10^−18^		6.2 × 10^−21^	3.5 × 10^−21^	6.5 × 10^−22^	6.2 × 10^−21^

k_1_ is the rate constant of Path 1. k_IM1-W1_, k_RW1_, k_RW2_ and k_RW3_ are the rate constants of Paths IM1-W1, RW1, RW2, and RW3, respectively.

## Supplementary Materials

The following are available online at http://www.mdpi.com/1420-3049/23/9/2240/s1. Table S1: Electronic energies (ΔE and Δ(E + ZPE)), enthalpies [(ΔH(298 K)], and Gibbs free energies [ΔG(298 K)] for the reaction with water monomer occurring through IM3 + H_2_O. Table S2: The equilibrium constants (in cm^3^ molecule^−1^) for the formation of CH_2_O···H_2_O, ClO···H_2_O, H_2_O···ClO, CH_2_O···(H_2_O)_2_ and ClO···(H_2_O)_2_ complexes at different altitudes. Table S3: The equilibrium constants (cm^3^ molecule^−1^ s^−1^) for the formation of IM1, IM2 and IM3 complexes at different altitudes. Table S4: Effective rate constants (cm^3^ molecule^−1^ s^−1^) for CH_2_O + ClO reaction with one water molecule at different altitudes (h). Table S5: The ratios of effective rate constants to corresponding rate constants for the CH_2_O + ClO reaction in the absence and presence of water at different heights. Table S6: Rate constants and corresponding effective rate constants (cm^3^ molecule^−1^ s^−1^) for the reactions in the presence of water dimer. Table S7 Cartesian coordinates of the optimized geometries at the B3LYP-D3/aug-cc-pVTZ level of theory. Table S8 ZPE corrected electronic energies of individual species with different methods. Figure S1: Optimized geometries for the hydrogen-bonded CH_2_O···H_2_O**,** ClO···H_2_O, H_2_O···ClO, CH_2_O···(H_2_O)_2_ and ClO···(H_2_O)_2_ complexes calculated at the B3LYP-D3/aug-cc-pVTZ level. Figure S2: Optimized geometries for the CH_2_O + ClO reaction without water calculated at the B3LYP-D3/aug-cc-pVTZ level. Figure S3: The energy profile of the CH_2_O + ClO reaction in the presence of water vapor occurring through IM3 + H_2_O pathway. Figure S4: Optimized geometries of the double hydrogen transfer path starting from ClO···H_2_O + CH_2_O pathway. Figure S5: The energy profile of the double hydrogen transfer path starting from ClO···H_2_O + CH_2_O pathway. Figure S6: The energy profile of the CH_2_O + ClO reaction in the presence of water dimer occurring through IM1 + (H_2_O)_2_ and IM2 + (H_2_O)_2_ pathways.
