# Supplementary material for: The Role of (H2O)1-2 in the CH2O + ClO Gas-Phase Reaction
Source: Molecules. 2018 Sep 3;23(9):2240. doi: 10.3390/molecules23092240 (PMC6225201; doi:10.3390/molecules23092240)
Supplement: Supplementary file 1 [file molecules-23-02240-s001.pdf]

Supplementary Materials

The role of $(H_2O)_{1-2}$ in the CH_2O + ClO gas-phase reaction

Junyao Li, Narcisse T. Tsona, and Lin Du*

Environment Research Institute, Shandong University, Binhai Road 72, 266237 Shandong, China; lijunyaoeri@mail.sdu.edu.cn (J.L.); tsonatch@sdu.edu.cn (N.T.)

* Correspondence: lindu@sdu.edu.cn; Tel.: +86-532-58631980

Table S1 Electronic energies (ΔE and Δ(E + ZPE)), enthalpies [(ΔH(298 K)], and Gibbs free energies [ΔG(298 K)] for the reaction with water monomer occurring through IM3 + H_2O. All energies are calculated relative to the energy of CH_2O + ClO + H_2O, in units of kcal mol^{-1}.

compound	ΔE	Δ(E+ZPE)	ΔH(298K)	ΔG(298K)
CH_2O+ClO+H_2O	0	0	0	0
IM3-W	-9.0	-6.5	-3.9	9.4
TS-IMc	6.6	9.1	10.4	27.3
IMc-W	-11.7	-8.0	-5.4	11.7
TS1W2	8.7	7.9	6.1	22.9
PC1W2	0.6	-1.1	-2.5	10.9
TS2W2	21.4	21.4	20.4	37.3
PC2W2	-77.3	-71.4	-66.8	-49.6
H+HCOOCl+H_2O	4.7	1.6	-1.6	1.5
Cl+HCOOH+H_2O	-60.5	-57.5	-54.9	-52.6

Table S2 The equilibrium constants (in $cm^3 \cdot molecule^{-1}$) for the formation of $CH_2O\cdots H_2O$, $ClO\cdots H_2O$, $H_2O\cdots ClO$, $CH_2O\cdots(H_2O)_2$ and $ClO\cdots(H_2O)_2$ complexes at different altitudes.

h(km)	T(K)	$[H_2O]_a$	K_{eq1}	K_{eq2}	K_{eq3}	K_{eq4}	K_{eq5}
0	298.15	7.7×10^{17}	2.2×10^{-23}	4.8×10^{-23}	6.4×10^{-23}	4.2×10^{-27}	1.5×10^{-27}
0	288.19	4.0×10^{17}	2.4×10^{-23}	5.0×10^{-23}	6.8×10^{-23}	5.9×10^{-27}	1.9×10^{-27}
2	275.21	1.9×10^{17}	2.7×10^{-23}	5.3×10^{-23}	7.6×10^{-23}	9.6×10^{-27}	2.6×10^{-27}
4	262.23	7.3×10^{16}	3.1×10^{-23}	5.7×10^{-23}	8.5×10^{-23}	1.6×10^{-26}	3.8×10^{-27}
6	249.25	2.6×10^{16}	3.6×10^{-23}	6.2×10^{-23}	9.7×10^{-23}	3.0×10^{-26}	5.5×10^{-27}
8	236.27	8.0×10^{15}	4.2×10^{-23}	6.8×10^{-23}	1.1×10^{-22}	5.7×10^{-26}	8.5×10^{-27}
10	223.29	2.1×10^{15}	5.1×10^{-23}	7.6×10^{-23}	1.3×10^{-22}	1.2×10^{-25}	1.4×10^{-26}
12	216.69	10.0×10^{14}	5.7×10^{-23}	8.1×10^{-23}	1.5×10^{-22}	1.8×10^{-25}	1.8×10^{-26}

K_{eq1}-K_{eq5} are the computed equilibrium constants for the formation of $CH_2O\cdots H_2O$, $ClO\cdots H_2O$ $H_2O\cdots ClO$, $CH_2O\cdots(H_2O)_2$ and $ClO\cdots(H_2O)_2$ complexes, respectively. [a]Water concentrations are taken from Ref 1. [1]

Table S3 The equilibrium constants ($cm^3 \cdot molecule^{-1} \cdot s^{-1}$) for the formation of IM1, IM2 and IM3 complexes at different altitudes.

h(km)	T(K)	$[H_2O]_a$	K_{eq}(IM1)	K_{eq}(IM2)	K_{eq}(IM3)
0	298.15	7.7×10^{17}	1.6×10^{-24}	3.5×10^{-25}	1.7×10^{-23}
0	288.19	4.0×10^{17}	1.6×10^{-24}	3.2×10^{-25}	1.8×10^{-23}
2	275.21	1.9×10^{17}	1.7×10^{-24}	2.8×10^{-25}	2.0×10^{-23}
4	262.23	7.3×10^{16}	1.7×10^{-24}	2.4×10^{-25}	2.3×10^{-23}
6	249.25	2.6×10^{16}	1.7×10^{-24}	2.0×10^{-25}	2.6×10^{-23}
8	236.27	8.0×10^{15}	1.8×10^{-24}	1.7×10^{-25}	3.0×10^{-23}
10	223.29	2.1×10^{15}	1.8×10^{-24}	1.4×10^{-25}	3.6×10^{-23}
12	216.69	10.0×10^{14}	1.9×10^{-24}	1.3×10^{-25}	4.0×10^{-23}

K_{eq}(IM1), K_{eq}(IM2), K_{eq}(IM3) are the computed equilibrium constants for the formation of binary complexes IM1, IM2 and IM3, respectively. [a]Water concentrations are taken from Ref 1.[1]

Table S4 Effective rate constants ($cm^3 \cdot molecule^{-1} \cdot s^{-1}$) for CH_2O + ClO reaction in the presence of one water molecule at different altitudes (h) in the earth atmosphere.

h(km)	T(K)	k_1	k'_{RW1}	k'_{RW2}	k'_{RW3}	$k'_{IM1\text{-}W1}$	$k'_{IM2\text{-}W1}$
0	298.15	2.6×10^{-16}	9.6×10^{-25}	2.4×10^{-25}	2.0×10^{-24}	5.2×10^{-26}	7.0×10^{-30}
0	288.19	1.8×10^{-16}	4.5×10^{-25}	1.1×10^{-25}	1.0×10^{-24}	2.4×10^{-26}	1.2×10^{-29}
2	275.21	1.1×10^{-16}	1.5×10^{-25}	3.9×10^{-26}	3.8×10^{-25}	8.3×10^{-27}	2.6×10^{-29}
4	262.23	6.5×10^{-17}	4.6×10^{-26}	1.2×10^{-26}	1.3×10^{-25}	2.5×10^{-27}	5.9×10^{-29}
6	249.25	3.6×10^{-17}	1.2×10^{-26}	3.1×10^{-27}	3.8×10^{-26}	6.8×10^{-28}	1.5×10^{-28}
8	236.27	1.8×10^{-17}	2.7×10^{-27}	7.1×10^{-28}	9.8×10^{-27}	1.5×10^{-28}	3.9×10^{-28}
10	223.29	8.8×10^{-18}	5.0×10^{-28}	1.3×10^{-28}	2.1×10^{-27}	2.9×10^{-29}	1.2×10^{-27}
12	216.69	5.9×10^{-18}	2.0×10^{-28}	5.2×10^{-29}	9.0×10^{-28}	1.2×10^{-29}	2.1×10^{-37}

k_1 is the rate constant of Path 1, k'_{RW1}, k'_{RW2}, $k'_{RW}3$ and $k'_{IM1\text{-}W1}$, $k'_{IM2\text{-}W1}$ are the effective rate constants of Paths RW1, RW2, RW3, RW4, IM1-W1 and IM2-W1, respectively.

Table S5 Ratios of effective rate constants to corresponding rate constants for the CH_2O + ClO reaction with and without water at different heights in the earth atmosphere.

h(km)	T(K)	k'_{RW1}/k_1	k'_{RW2}/k_1	k'_{RW3}/k_1	$k'_{IM1\text{-}W1}/k_1$	$k'_{IM2\text{-}W2}/k_2$
0	298.15	3.7×10^{-9}	9.3×10^{-10}	7.8×10^{-9}	2.0×10^{-10}	3.2×10^{-7}
0	288.19	2.4×10^{-9}	6.2×10^{-10}	5.5×10^{-9}	1.3×10^{-10}	2.2×10^{-7}
2	275.21	1.4×10^{-9}	3.5×10^{-10}	3.4×10^{-9}	7.4×10^{-11}	1.3×10^{-7}
4	262.23	7.0×10^{-10}	1.8×10^{-10}	2.0×10^{-9}	3.9×10^{-11}	7.6×10^{-8}
6	249.25	3.4×10^{-10}	8.8×10^{-11}	1.1×10^{-9}	1.9×10^{-11}	4.0×10^{-8}
8	236.27	1.5×10^{-10}	3.9×10^{-11}	5.3×10^{-10}	8.4×10^{-12}	1.9×10^{-8}
10	223.29	5.7×10^{-11}	1.5×10^{-11}	2.4×10^{-10}	3.3×10^{-12}	8.5×10^{-9}
12	216.69	3.4×10^{-11}	8.9×10^{-12}	1.5×10^{-10}	2.0×10^{-12}	5.3×10^{-9}

Table S6 Rate constants and corresponding effective rate constants ($cm^3 \cdot molecule^{-1} \cdot s^{-1}$) for the reactions with a water dimer inclusion.

h(km)	T(K)	$[H_2O]_2$	k_1	k_{RWW1}	k_{RWW2}	k'_{RWW1}	k'_{RWW2}
0	298.15	3.1×10^{14}	2.6×10^{-16}	1.9×10^{-23}	1.1×10^{-24}	2.5×10^{-35}	5.1×10^{-37}
0	288.19	1.2×10^{14}	1.8×10^{-16}	2.0×10^{-23}	1.0×10^{-24}	1.3×10^{-35}	2.3×10^{-37}
2	275.21	2.8×10^{13}	1.1×10^{-16}	2.0×10^{-23}	9.6×10^{-25}	5.4×10^{-36}	7.0×10^{-38}
4	262.23	5.7×10^{12}	6.5×10^{-17}	2.1×10^{-23}	9.0×10^{-25}	1.9×10^{-36}	1.9×10^{-38}
6	249.25	1.3×10^{10}	3.6×10^{-17}	2.1×10^{-23}	8.4×10^{-25}	6.1×10^{-37}	4.5×10^{-39}
8	236.27	9.6×10^{11}	1.8×10^{-17}	2.2×10^{-23}	7.8×10^{-25}	1.7×10^{-37}	8.6×10^{-40}
10	223.29	1.3×10^{10}	8.8×10^{-18}	2.4×10^{-23}	7.3×10^{-25}	3.7×10^{-38}	1.3×10^{-40}
12	216.69	3.7×10^{9}	5.9×10^{-18}	2.4×10^{-23}	7.0×10^{-25}	1.6×10^{-38}	4.7×10^{-41}

k_{RWW1}, k_{RWW2} are the rate constants of Path RWW1 and Path RWW2, respectively.
k'_{RWW1}, k'_{RWW2} are the effective rate constants of Path RWW1 and Path RWW2, respectively.

Table S7 Cartesian coordinates of the optimized geometries at the B3LYP-D3/aug-cc-pVTZ level of theory.

CH_2O

C	0.00000000	-0.52697800	0.00000000
H	0.93839200	-1.11325700	0.00000000
H	-0.93839200	-1.11325700	0.00000000
O	0.00000000	0.67354800	0.00000000

ClO

Cl	0.00000000	0.00000000	0.50894700
O	0.00000000	0.00000000	-1.08151200

H_2O

O	0.00000000	0.00000000	0.11698300
H	0.00000000	0.76357100	-0.46793300
H	0.00000000	-0.76357100	-0.46793300

$(H_2O)_2$

O	-1.51085600	-0.00035800	-0.12133700
H	-1.92655100	0.00240700	0.74489600
H	-0.55683200	0.00163600	0.05156100
O	1.38936100	0.00053100	0.11224800
H	1.72460400	-0.76904300	-0.35882900
H	1.73073800	0.76361600	-0.36491600

IM1

C	1.99298500	0.46146100	0.00033500
H	1.14305100	1.17253700	0.00206600
H	3.00626800	0.90397600	-0.00078800
O	1.82264300	-0.72666600	-0.00030000
Cl	-1.31024500	-0.46377400	0.00012700
O	-1.05177500	1.10652600	-0.00038100

IM2

C	-1.77391500	-0.11299700	0.00002800
H	-1.12813100	0.78963200	0.00006800
H	-1.22992400	-1.08006100	0.00002900
O	-2.96939700	-0.05287600	-0.00002200
Cl	1.66749400	-0.41602600	-0.00000300
O	1.05116400	1.05798200	-0.00000400

IM3

C	-2.41893400	0.50741600	-0.00001400
H	-1.81236100	1.43082900	0.00037200
H	-3.51570500	0.63601600	-0.00049300
O	-1.91077600	-0.58275300	0.00008200
Cl	0.91814200	-0.16937800	-0.00004300
O	2.43993300	0.30376400	0.00003400

IM1-W

C	-2.07510100	-1.33605300	-0.10203400
H	-1.00436600	-1.38972400	-0.35723100
H	-2.63731600	-2.28494300	-0.09031200
O	-2.61331500	-0.28810100	0.15660100
Cl	1.43710000	0.16689200	-0.00308100
O	2.59658100	-0.92777200	0.05473500
O	-0.73159700	1.84127300	-0.13067500
H	-0.86558200	2.60149500	0.44138400
H	-1.48617100	1.24912200	0.02545600

IM2-W

C	-0.92689900	0.91981600	0.00760700
H	-0.03324600	1.57000900	0.01201700
H	-0.74569200	-0.16785700	-0.05634300
O	-2.04145400	1.36961300	0.06921900
Cl	2.18597500	-0.73313600	0.04863100
O	2.20949500	0.85104400	-0.11969100
O	-3.12220900	-1.29025200	-0.03836400
H	-3.15329800	-0.32366200	0.00891600
H	-4.03459700	-1.57732900	-0.12626800

$CH_2O\cdots H_2O$

C	1.30623900	0.46515800	0.00083000
H	2.38323500	0.70709700	0.00062200
H	0.60052100	1.31233300	0.00224900
O	0.92051900	-0.67679500	-0.00050300
O	-1.81385400	0.17663600	-0.00227600
H	-1.06879400	-0.44248500	-0.00076100
H	-2.60571500	-0.36662400	0.01514800

IM1W1

C	0.96186100	0.90738500	0.00008200
H	0.00069300	1.45324100	0.00014100
H	1.88753300	1.50583300	-0.00005500
O	0.99478800	-0.29745200	0.00008500
O	3.87148500	-0.19406100	0.00000100
H	3.01263700	-0.63973700	0.00004300
H	4.53051900	-0.89281900	-0.00060500
Cl	-2.16185600	-0.62640300	0.00000700
O	-2.17264800	0.96376500	-0.00010400

$ClO\cdots H_2O$

Cl	1.26605300	-0.32970200	0.00025400
O	0.31906200	0.94493600	-0.00032500
O	-2.41262800	-0.31218300	-0.00085800
H	-1.62171100	0.24192900	-0.00325800
H	-3.15265600	0.30098500	0.00840000

IM2W1

Cl	-2.04695100	-0.38263300	-0.27241400
O	-0.96398000	-0.11556600	0.88711200
O	0.76362400	1.99164900	-0.17996100
H	0.08335400	1.60399600	0.38694100
H	1.20622800	2.65549500	0.35628100
C	1.72916700	-0.65720400	-0.30443800
H	1.58600500	-0.31749900	-1.34534600
H	0.79914700	-0.68568600	0.31447700
O	2.79391100	-0.97712300	0.13601400

$H_2O\cdots ClO$

Cl	-0.47763900	0.00030800	-0.02041500
O	-2.06951900	-0.00040500	0.03546800
O	2.37710200	-0.00006600	-0.06692700
H	2.83040100	0.76509300	0.29977500
H	2.82880600	-0.76656100	0.29895800

IM2W2

Cl	1.43710800	0.16702800	-0.00304700
O	2.59662500	-0.92759900	0.05461600
O	-0.73197000	1.84114000	-0.13054700
H	-1.48661500	1.24901900	0.02526900
H	-0.86628000	2.60154900	0.44118200
C	-2.07461200	-1.33612000	-0.10184900
H	-2.63644300	-2.28524500	-0.09031500
H	-1.00370200	-1.38946000	-0.35641200
O	-2.61342000	-0.28836900	0.15632800

$CH_2O\cdots(H_2O)_2$

C	1.60922800	-0.77009000	-0.00596200
H	0.65689000	-1.32049200	-0.04108200
H	2.54326100	-1.35781900	0.02209300
O	1.64562100	0.43907000	0.00263800
O	-1.63748200	-1.22608700	0.08426500
H	-2.38408200	-1.48929500	-0.45960600
H	-1.59593700	-0.25305800	0.02539300
O	-0.97228700	1.49001500	-0.09505300
H	-0.01697600	1.29701200	-0.05525900
H	-1.14534400	2.12020800	0.60943000

$ClO\cdots(H_2O)_2$

Cl	1.01642000	0.24878500	-0.01218300
O	2.38338100	-0.57396900	0.01837200
O	-1.48860300	1.44641400	-0.09755000
H	-1.93596100	0.58949600	0.00301900
H	-1.79950800	1.99655500	0.62646500
O	-2.11804600	-1.30790000	0.09924400
H	-2.57285900	-1.79780900	-0.59268400
H	-1.18467000	-1.53394100	0.00978300

IMWW1

C	-0.36906700	-0.79647700	-0.00591700
H	-1.33203300	-1.32977400	-0.02036200
H	0.56020800	-1.39515100	0.01020000
O	-0.31560800	0.41183200	-0.00853200
O	-3.59719700	-1.20603400	0.05249400
H	-4.32313000	-1.46522200	-0.52058300
H	-3.56170500	-0.23206500	0.01418400
O	-2.92909400	1.51494200	-0.05634400
H	-1.97637100	1.31276400	-0.03022800
H	-3.09289600	2.11813500	0.67367300
Cl	2.86612400	0.50641600	-0.03577500
O	2.74392500	-1.07560200	0.07698100

IMWW2

Cl	0.96410600	-0.96588200	0.16210300
O	1.81312700	-2.25815700	-0.24562000
O	-0.41409400	1.24304200	0.77074900
H	0.20144300	1.86627100	0.35599400
H	-1.24560300	1.29608400	0.27635400
O	1.91266800	2.38562400	-0.44173300
H	2.52053600	2.96289900	0.03016800
H	2.31413400	1.50936500	-0.41802100
C	-2.91088900	-0.64554700	-0.08064300
H	-3.75317600	-1.32051300	-0.31048500
H	-2.05737200	-1.07805600	0.46734000
O	-2.92475500	0.51164300	-0.41755100

Table S8 ZPE corrected electronic energies of individual species with different methods.

	B3LYP-D3/aug-cc-pVTZ	CCSD(T)/aug-cc-pVTZ
CH_2O	-114.5260934	-114.3163046
ClO	-535.3330406	-534.7459781
H_2O	-76.4449697	-76.3210675
$(H_2O)_2$	-152.8949676	-152.6470296
IM1	-649.8998327	-649.0649102
IM2	-649.8982715	-649.0617391
IM3	-649.9000708	-649.0668856
IM1-W	-726.3530071	-725.3955847
IM2-W	-726.3490404	-725.3908083
$CH_2O\cdots H_2O$	-190.9767223	-190.6431829
IM1W1	-726.3503672	-725.3922124
$ClO\cdots H_2O$	-611.8182165	-611.0708309
IM2W1	-726.3486748	-725.3870967
$H_2O\cdots ClO$	-611.8187577	-611.0714715
IM2W2	-726.3530071	-725.3955847
$CH_2O\cdots(H_2O)_2$	-267.4323846	-266.9742547
$ClO\cdots(H_2O)_2$	-688.271151	-687.3993747
IMWW1	-802.8060616	-801.723293
IMWW2	-802.8039919	-801.7222533

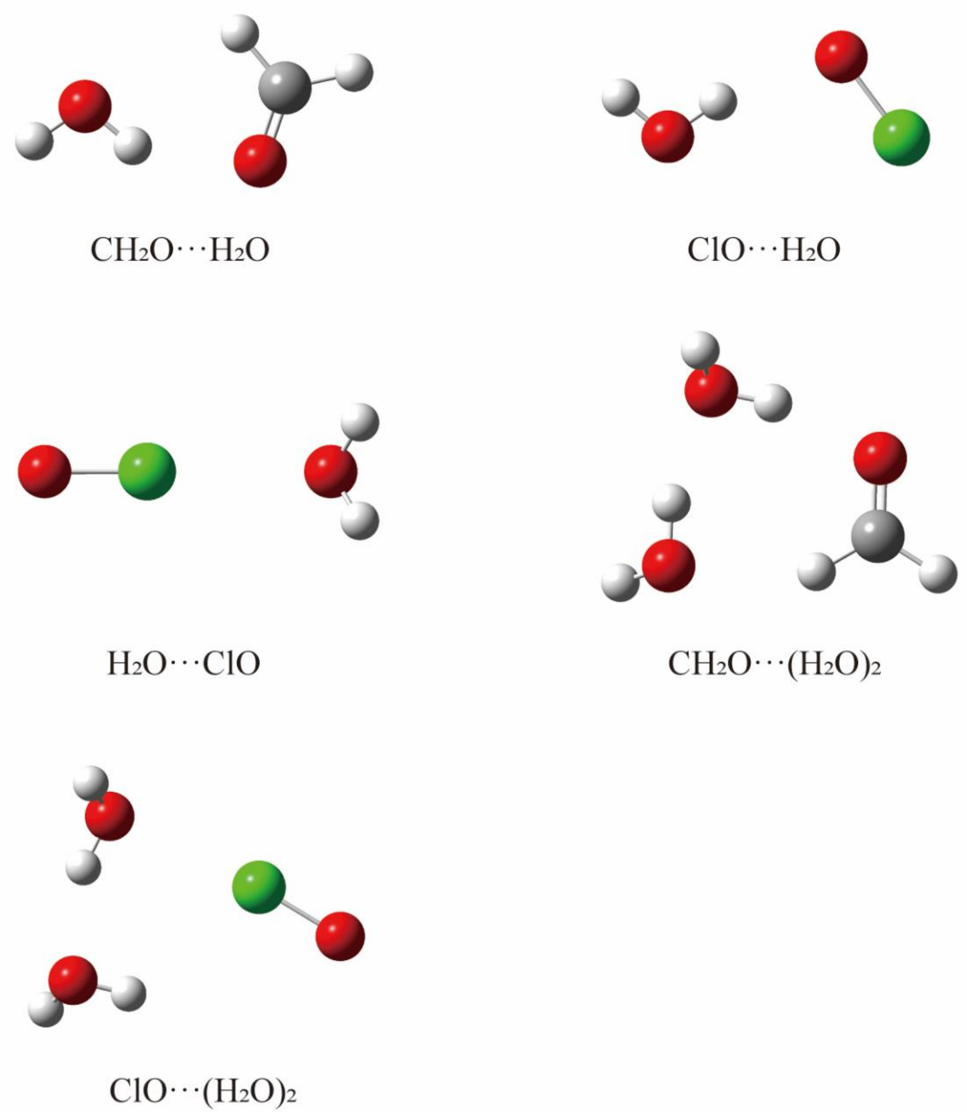

Figure S1 Optimized geometries for the hydrogen-bonded $CH_2O\cdots H_2O$, $ClO\cdots H_2O$, $H_2O\cdots ClO$, $CH_2O\cdots(H_2O)_2$ and $ClO\cdots(H_2O)_2$ complexes calculated at the B3LYP-D3/aug-cc-pVTZ level of theory. The atoms in green, white and red color denote Cl atom, H atom and O atom, respectively.

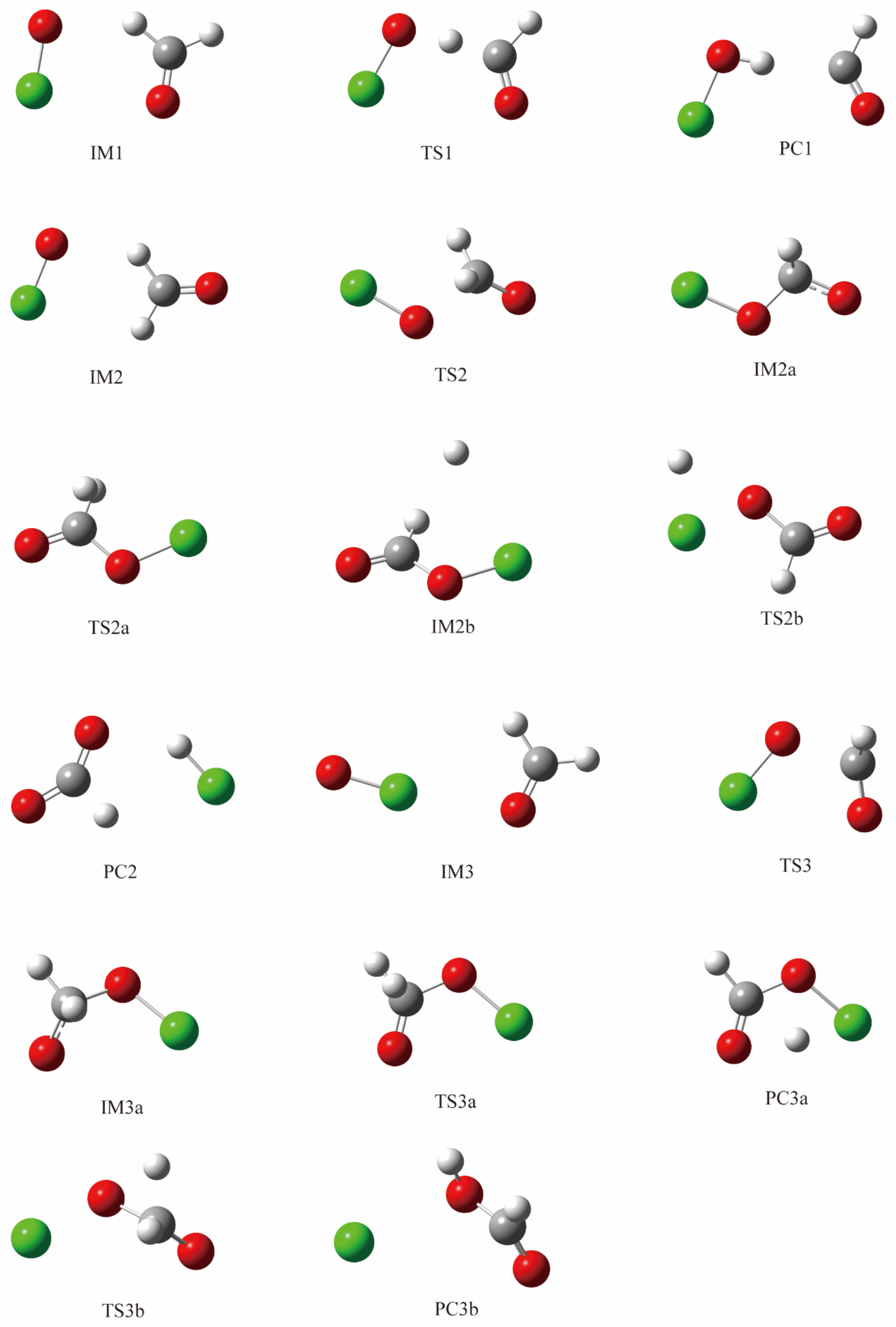

Figure S2 Optimized geometries for the CH_2O + ClO reaction without water calculated at the B3LYP-D3/aug-cc-pVTZ level of theory. The atoms in green, white and red color denote Cl atom, H atom and O atom, respectively.

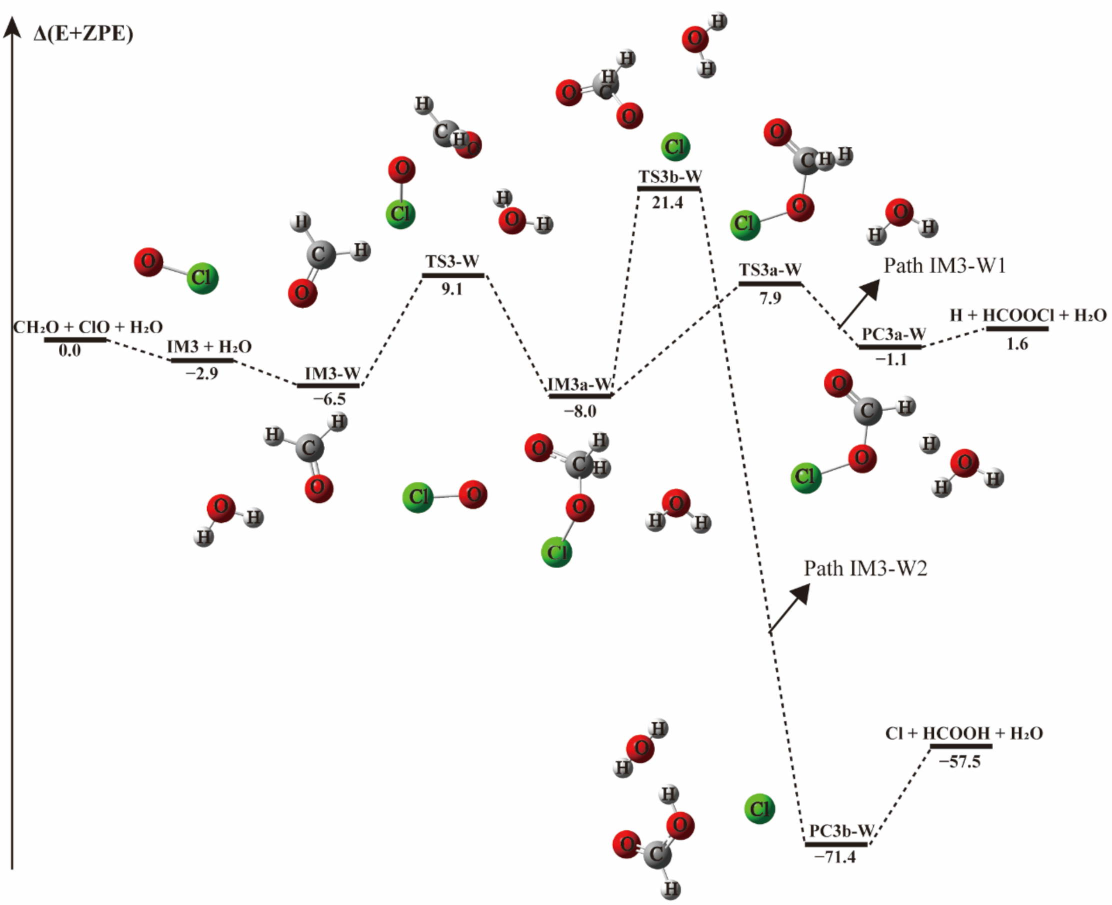

Figure S3 The energy profile of the CH_2O + ClO reaction in the presence of water vapor occurring through IM3 + H_2O pathway. Energies (in kcal mol^{-1}) are calculated at the CCSD(T)/aug-cc-pVTZ//B3LYP/aug-cc-pVTZ level.

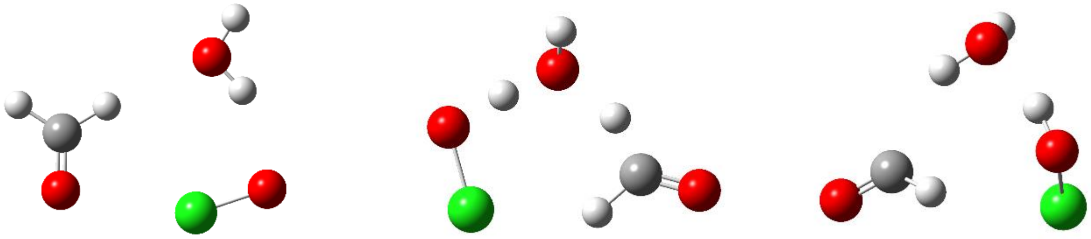

IM2W3 TS2W3 PC2W3

Figure S4 Optimized geometries of the double hydrogen transfer path starting from ClO···H_2O + CH_2O pathway. The atoms in green, white and red color denote Cl atom, H atom and O atom, respectively.

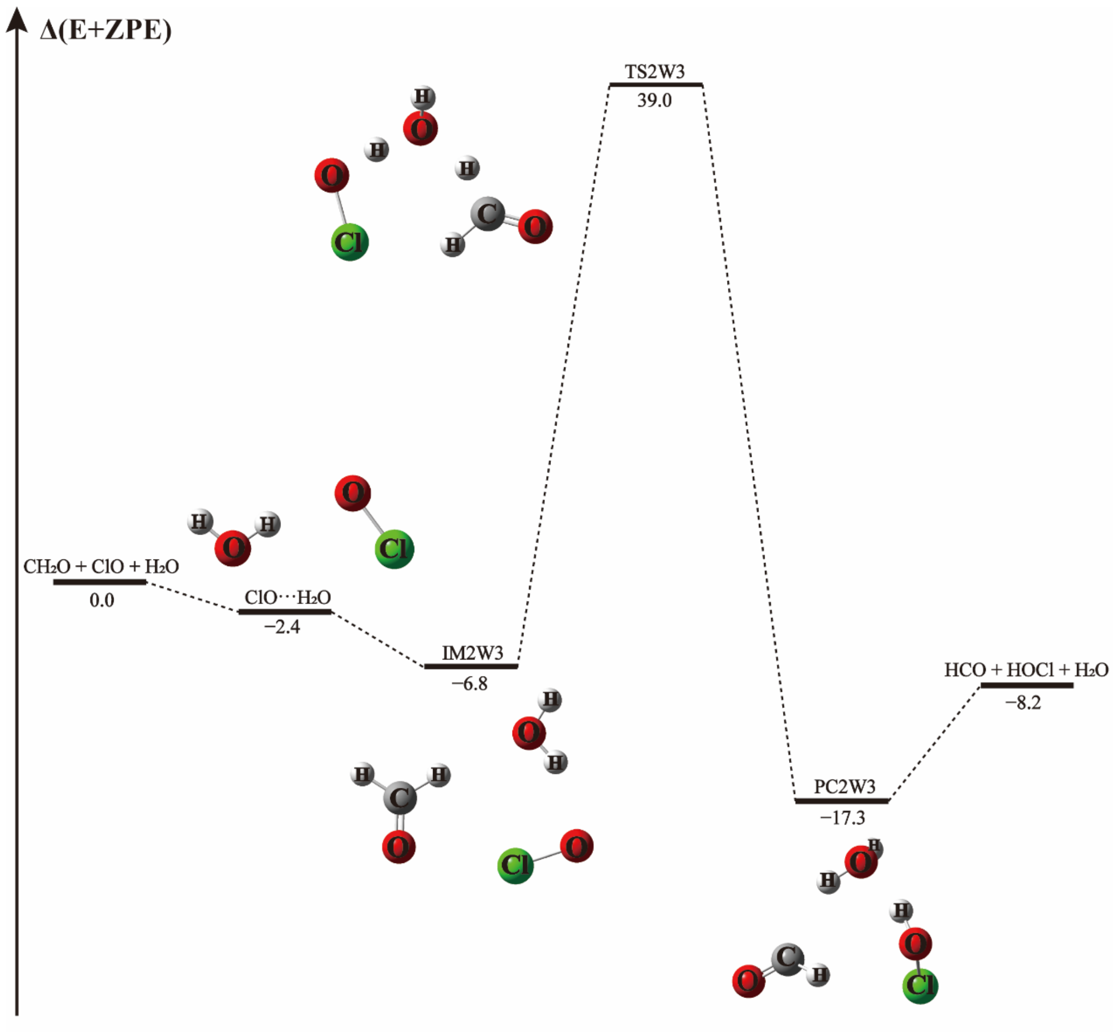

Figure S5 The energy profile of the double hydrogen transfer path starting from $ClO\cdots H_2O$ + CH_2O pathway. Energies (in kcal mol^{-1}) are calculated at the CCSD(T)/aug-cc-pVTZ//B3LYP/aug-cc-pVTZ level.

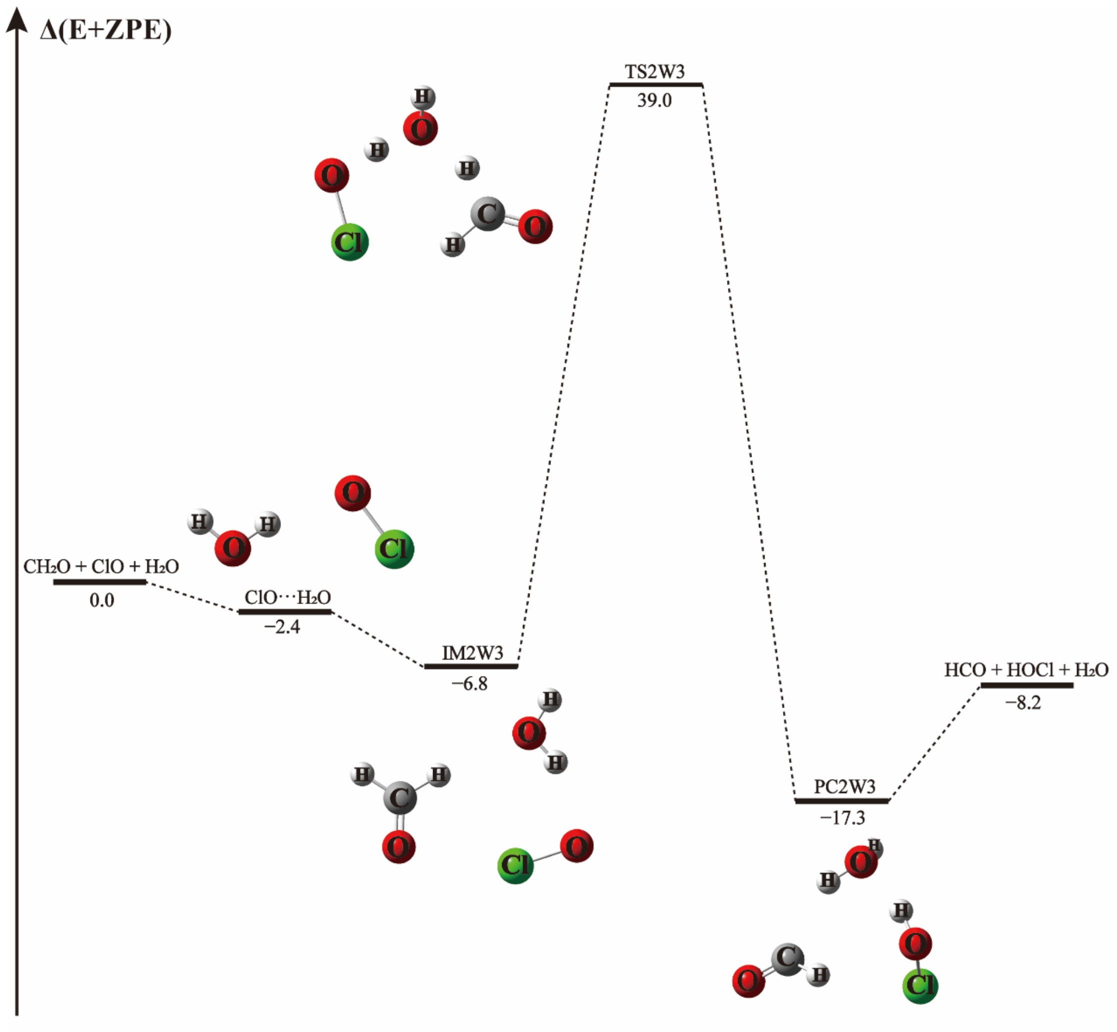

Figure S6 The energy profile of the CH_2O + ClO reaction in the presence of water dimer occurring through IM1 + $(H_2O)_2$ and IM2 + $(H_2O)_2$ pathways. Energies (in kcal mol^{-1}) are calculated at the CCSD(T)/aug-cc-pVTZ//B3LYP/aug-cc-pVTZ level.

Reference:

1. Lowe, P. R. An approximating polynomial for the computation of saturation vapor pressure. *J. Appl. Meteorol.* **1977,** 16, 100-103.
